# Construction of an Amethyst-like MoS_2_@Ni_9_S_8_/Co_3_S_4_ Rod Electrocatalyst for Overall Water Splitting

**DOI:** 10.3390/nano13162302

**Published:** 2023-08-10

**Authors:** Zhen Pei, Tengteng Qin, Rui Tian, Yangxin Ou, Xingzhong Guo

**Affiliations:** 1 State Key Laboratory of Silicon and Advanced Semiconductor Materials, School of Materials Science and Engineering, Zhejiang University, Hangzhou 310058, China; 22126097@zju.edu.cn (Z.P.); 12226085@zju.edu.cn (T.Q.); 22026092@zju.edu.cn (R.T.); 22226070@zju.edu.cn (Y.O.); 2 Hangzhou Global Scientific and Technological Innovation Center, Zhejiang University, Hangzhou 311200, China

**Keywords:** MoS_2_@Ni_9_S_8_/Co_3_S_4_, amethyst-like rods, bifunctional electrocatalysts, overall water splitting

## Abstract

Transition metal sulphide electrocatalytic materials possess the bright overall water-splitting performance of practical electrocatalytic technologies. In this study, an amethyst-like MoS_2_@Ni_9_S_8_/Co_3_S_4_ rod electrocatalyst was constructed via a one-step hydrothermal method with in-situ-grown ZIF-67 nanoparticles on nickel foam (NF) as a precursor. The rational design and synthesis of MoS_2_@Ni_9_S_8_/Co_3_S_4_ endow the catalyst with neat nanorods morphology and high conductivity. The MoS_2_@Ni_9_S_8_/Co_3_S_4_/NF with the amethyst-like rod structure exposes abundant active sites and displays fast electron-transfer capability. The resultant MoS_2_@Ni_9_S_8_/Co_3_S_4_/NF exhibits outstanding hydrogen evolution reaction (HER) and oxygen evolution reaction (OER) electrocatalytic activities, with low overpotentials of 81.24 mV (HER) at 10 mA cm^−2^ and 159.67 mV (OER) at 50 mA cm^−2^ in 1.0 M KOH solution. The full-cell voltage of overall water splitting only achieves 1.45 V at 10 mA cm^−2^. The successful preparation of the amethyst-like MoS_2_@Ni_9_S_8_/Co_3_S_4_ rod electrocatalyst provides a reliable reference for obtaining efficient electrocatalysts for overall water splitting.

## 1. Introduction

Studies have long focused on developing environmentally friendly and recyclable clean energy materials to replace depleted fossil fuels [1,2,3]. In this respect, hydrogen has become one of the most important material candidates for future energy technologies, owing to its cleanliness, renewability, and high calorific value [4,5,6]. Compared with numerous other hydrogen-production methods (e.g., coal gasification, steam methane reforming, biomass conversion, and photocatalytic hydrogen [7]), water-splitting technology has more potential since it only needs a certain voltage to produce high-purity hydrogen simply and without any pollution [8]. Typically, water splitting produces hydrogen and oxygen via the hydrogen evolution reaction (HER) and oxygen evolution reaction (OER) [9]. In the alkaline solution, the HER usually occurs through Volmer–Heyrovsky or Volmer–Tafel mechanism as follows [10,11]:

$$H_{2}O+e^{-}+*\to H^{*}+{OH}^{-}\left(Volmer\;step\right)$$

$$H_{2}O+e^{-}+H^{*}\to H_{2}+{OH}^{-}+*\left(Heyrovsky\;Step\right)$$

$$H^{*}+H^{*}\to H_{2}+2*(Tafel\;step)$$

wherein ∗ is the active adsorption site of the catalyst and ($H^{*}$) is the adsorbed hydrogen. The OER mechanism can be described as follows [12]:

$${OH}^{-}+*\to {OH}^{*}+e^{-}$$

$${OH}^{*}+{OH}^{-}\to O^{*}+H_{2}O+e^{-}$$

$$O^{*}+{OH}^{-}\to {OOH}^{*}+e^{-}$$

$${OOH}^{*}+{OH}^{-}\to O_{2}+*+H_{2}O+e^{-}$$

wherein ∗ is the active adsorption site, and ${OH}^{*}$, $O^{*}$, and ${OOH}^{*}$ are the intermediates. Compared with the HER, the OER involves more intermediates and has a more complicated reaction process. Due to the confinement of material active sites and the complex electron-transfer process of the OER, a high potential is typically required to drive the actual water splitting [13]. Electrocatalysts based on noble metals (Pt and Ru/Ir) can effectively reduce the overpotential of the HER and OER in the water-splitting process. However, the rare reserves and high cost of noble-metal-based electrocatalysts prevent them from being applied to long-term and large-scale hydrogen production in the future [14,15,16]. Therefore, the development of low-cost bifunctional non-noble metal electrocatalysts with high activity is key to achieving long-term and efficient hydrogen production through water splitting [17,18,19].

Earth-abundant transition metals have attracted considerable attention because of their good catalytic activity and low cost [11,12]. To date, numerous transition-metal-based electrocatalytic materials have been studied, including Ni-based sulphides [10,20], Co-based phosphides/sulphides [8,21,22,23], and Mo-based sulphides [24,25]. Among these materials, Ni-, Co-, and Mo-based sulphides are considered potential electrocatalysts for water splitting because of their abundant active sites and adjustable electronic properties [26]. However, single-component sulphide materials typically exhibit monofunctional catalytic activity for either the hydrogen evolution reaction (HER) or oxygen evolution reaction (OER). Strategies by combining different kinds of transition metal sulphides can effectively improve the activity of catalysts and achieve bifunctional catalytic performance [27,28,29]. Chen et al. constructed a Ni_9_S_8_/MoS_2_ nanosheet-modified NiMoO_4_ nanorod electrocatalyst with overpotentials of 190 (HER) and 360 mV (OER) at a current density of 10 mA cm^−2^ [27]. Yang et al. synthesized a hierarchical nanoassembly MoS_2_/Co_9_S_8_/Ni_3_S_2_/Ni electrocatalyst, which exhibited high HER and OER bifunctional catalytic activities in a wide pH range [30].

Moreover, designing and regulating the structural morphology of electrocatalytic materials are also effective strategies to enhance the activity of electrocatalysts [21]. Recently, the construction of unique electrocatalyst morphologies by incorporating zeolitic imidazolate frameworks (ZIFs) has attracted significant interest [24,31,32,33]. The introduction of ZIF-67 during the preparation of electrocatalysts can not only regulate the structure of the electrocatalyst but also introduce the Co element [33,34,35,36,37,38,39,40]. For example, Guo et al. synthesized hollow Co_3_S_4_@MoS_2_ derived from ZIF-67 using a two-step hydrothermal and calcination method, whereby hollow Co_3_S_4_@MoS_2_ exhibited an overpotential of 330 mV (HER) at 10 mA cm^−2^ [41]. Peng et al. combined carbon nanofibers and ZIF-67 to prepare tubular electrocatalytic materials with rough surfaces that exhibited enhanced catalytic activity for the OER after calcination [42]. However, most electrocatalytic materials derived from ZIF-67 exhibited high overpotentials, owing to the poor conductivity of ZIF-67 [13,24]. Although high-temperature calcination can enhance the conductivity of electrocatalysts, it may destroy the structural characteristics and active catalytic sites of the catalyst [43,44]. According to the previous reports, ZIF-67 can be etched by the sulphide to release Co^2+^ under hydrothermal conditions, and the conductivity and exposed active sites of the ZIF-derived electrocatalysts can be improved [45]. However, most of the research directly added ZIF-67 powder into the precursor solution to regulate reactions, which may lead to the serious agglomeration of the prepared electrocatalyst [33,46]. The preparation of electrocatalysts by a one-step hydrothermal method using ZIF-67/NF as a precursor is rarely reported. Ge et al. reported a non-agglomerated MoS_2_/CoP electrocatalyst exhibiting enhanced HER activity prepared by hydrothermal reaction of ZIF-67 precursor grown on titanium foil (TF) [47]. 

Herein, we demonstrate the preparation of an amethyst-like MoS_2_@Ni_9_S_8_/Co_3_S_4_ rod electrocatalyst with high conductivity and rich active sites via a convenient hydrothermal method. Benefiting from the synergistic effects and improved electronic environment, MoS_2_@Ni_9_S_8_/Co_3_S_4_/NF shows low overpotentials for the HER at 10 mA cm^−2^ and the OER at 50 mA cm^−2^ in alkaline solution and possesses a low full-cell voltage of overall water splitting at 10 mA cm^−2^ and excellent electrocatalytic stability. 

## 2. Materials and Methods

### 2.1. Materials

Nickel foam (0.5 mm thickness) was purchased from Saibo Electrochemical Materials (China). Co(NO_3_)_2_·6H_2_O, Na_2_MoO_4_·2H_2_O, 2-methylimidazole (C_4_H_6_N_2_), and thioacetamide (C_2_H_5_NS) were purchased from Aladdin Chemical Reagent. Poly(sodium-p-styrene sulfonate) (C_8_H_7_NaO_3_S)_n_ was purchased from Macklin Biochemical Reagent, and methanol (CH_3_OH) was purchased from Sinopharm Chemical Reagent. All the reagents and chemicals were used without further purification.

### 2.2. Preparation of the ZIF-67/NF Precursor

The ZIF-67 precursor was directly grown on NF substrate via a simple process according to a previously reported method with some modifications [42]. First, NF (2 × 3 cm^2^) was cleaned with 3 M HCl solution, deionized water, and ethanol for 10 min under ultrasonication in successive order and dried at 70 °C for 12 h. Next, 40 mg poly(sodium-p-styrene sulfonate) was added to 10 mL deionized water to form solution I. To achieve surface modification, the as-cleaned NF was soaked in solution I for 30 min under ultrasonication and then washed thrice with deionized water. Next, 40 mmol 2-methylimidazole (2-MeIM) was distributed in 50 mL methanol to form solution II, and 5 mmol Co(NO_3_)_2_·6H_2_O was added to 50 mL methanol to form solution III. The surface-modified NF was fully immersed in solution II for 30 min. Subsequently, solution III was poured into the resulting solution II with the surface-modified NF solution mixture and agitated for 30 min. The obtained blend was then allowed to stand at room temperature (25 °C) for 24 h. The desired product was obtained and washed thrice with deionized water. Finally, the product was dried at 70 °C in a vacuum oven for 12 h and named ZIF-67/NF.

### 2.3. Synthesis of Amethyst-like MoS_2_@Ni_9_S_8_/Co_3_S_4_ Rods

First, 1 mmol Na_2_MoO_4_·2H_2_O and 3 mmol thioacetamide (C_2_H_5_NS) were dissolved in 40 mL deionized water. The resultant solution was agitated for 30 min, after which one piece of the as-prepared ZIF-67/NF and the above solution was transferred into a 50 mL autoclave, where it was maintained at 180 °C for 6 h. Subsequently, the resultant sample was collected and washed several times with ethanol and deionized water. Finally, the amethyst-like MoS_2_@Ni_9_S_8_/Co_3_S_4_ rod electrocatalyst was obtained after drying at 70 °C in a vacuum oven for 12 h. For comparison, MoS_2_/Ni_9_S_8_/NF without a ZIF-67 precursor, Mo-doped ZIF-67/NF without an S source, and S-doped ZIF-67/NF without a Mo source were synthesized using a preparation process similar to that of the amethyst-like MoS_2_@Ni_9_S_8_/Co_3_S_4_ rods.

### 2.4. Material Characterization

The crystalline structures of all materials were characterized using a PANalytical X’Pert X-ray diffractometer (bruker D8 ADVACNCE, Bruker, Mannheim, Germany) with a Cu Kα radiation source at 30 kV. The morphologies and structures of the samples were observed using a Gemini SEM 300 scanning electron microscope (SEM) (Gemini, Friedrichshafen, Germany) and a JEOL JEM-2100F transmission electron microscope (JEOL Co., Ltd., Tokyo, Japan). X-ray photoelectron spectroscopy was conducted using a Thermo Scientific K-Alpha instrument (Waltham, MA, USA).

### 2.5. Electrochemical Measurements

All electrochemical tests were performed using a three-electrode system in a 1 M KOH electrolyte on a CHI 660E electrochemical workstation (CHI 660E, Shanghai, China). The as-prepared catalyst (1 × 1 cm^2^) was used as the working electrode, and a graphite rod and Hg/HgO were used as the counter and reference electrodes, respectively. All potentials were converted to the reversible hydrogen electrode (RHE) potential according to the following equation: $E_{RHE}=E_{Hg/HgO}+0.0591pH+0.098$ [18]. Linear sweep voltammetry (LSV) was performed from −1.85 to −0.70 V (−0.20 to 1.40 V) vs. Hg/HgO for the hydrogen evolution reaction (HER) (oxygen evolution reaction (OER)) at a scan rate of 2 mV/s. Electrochemical impedance spectroscopy (EIS) was performed in the frequency range of 100 KHz to 0.01 Hz at −1.03 V vs. Hg/HgO (0.56 V) for the HER (OER). The double-layer capacitance (C_dl_) and electrochemical surface area (ECSA) were obtained using cyclic voltammetry (CV) at scan rates of 20–100 mV s^−1^ in the range of −0.30 to −0.20 V vs. Hg/HgO. All polarization curves were corrected using iR compensation.

## 3. Results

**Figure 1:**
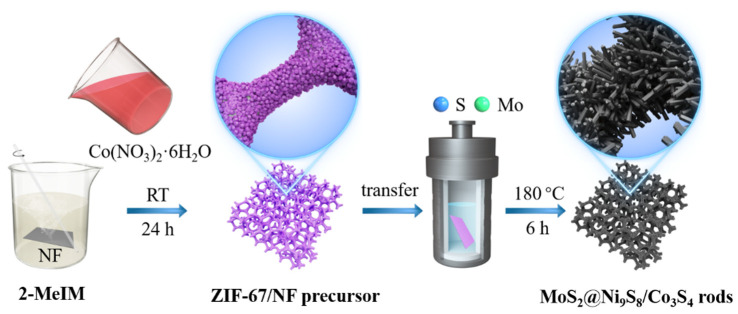
Schematic of the synthesis process of the amethyst-like MoS_2_@Ni_9_S_8_/Co_3_S_4_ rod electrocatalyst.

**Figure 2:**
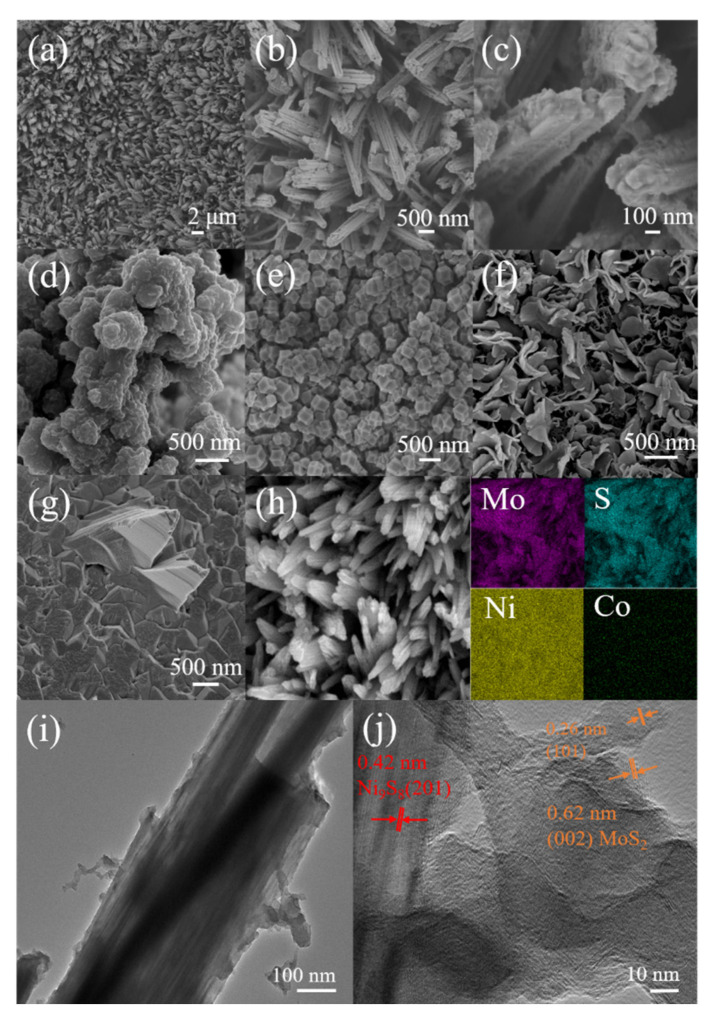
SEM images of (**a**–**c**) MoS_2_@Ni_9_S_8_/Co_3_S_4_/NF, (**d**) MoS_2_/Ni_9_S_8_/NF, (**e**) ZIF-67, (**f**) Mo-ZIF-67/NF, and (**g**) S-ZIF-67/NF. (**h**) EDS elemental mapping, (**i**) TEM, and (**j**) HRTEM images of MoS_2_@Ni_9_S_8_/Co_3_S_4_/NF.

An amethyst-like MoS_2_@Ni_9_S_8_/Co_3_S_4_ rod electrocatalyst was synthesized via a one-pot hydrothermal reaction (Figure 1). A precursor comprising ZIF-67 deposited on NF was first obtained by the hydrothermal reaction at room temperature. As shown in Figure 2e, the prepared ZIF-67/NF precursor presented a classical uniform polyhedral morphology with a size range of 100–300 nm [48]. Subsequently, in the hydrothermal process, the ZIF-67/NF immersed in the solution first reacted with sulphides to release Co ions and generate Co_3_S_4_, resulting in the structural evolution of ZIF-67/NF [45]. Compared with S-ZIF-67/NF (Figure 2g), it can be seen that the structure of ZIF-67/NF became dense with sharp protrusions after reacting with sulphides, which may have a good structure-orienting effect for the further formation of nanorods on it. Then, the Ni from the NF reacted with sulphides to generate Ni_9_S_8_ nanorods, and Mo reacted with sulphides to form MoS_2_ [46,47]. As the reaction proceeded, eventually, an amethyst-like MoS_2_@Ni_9_S_8_/Co_3_S_4_ rod electrocatalyst was successfully obtained via this one-step hydrothermal method. 

Figure 2 shows SEM images of the different samples. The microstructures of MoS_2_@Ni_9_S_8_/Co_3_S_4_/NF at different magnifications are shown in Figure 2a–c. A uniform structure with a neat nanorods array of MoS_2_@Ni_9_S_8_/Co_3_S_4_/NF is shown in Figure 2a, whereby the structural characteristics of a single nanorod are similar to those of an amethyst-like rod (Figure 2b). Each nanorod presents a rough surface and polygonal morphology, with a diameter in the range of 200–500 nm (Figure 2c). ZIF-67 particles disappear compared with the precursor before sulphuration, indicating that ZIF-67 completely reacted with sulphides. The non-agglomerated rod-shaped structure of MoS_2_@Ni_9_S_8_/Co_3_S_4_/NF can expose rich active sites and facilitate the detachment of bubbles from the catalyst during water splitting [27,49]. The morphologies of the other comparative samples are significantly different from that of MoS_2_@Ni_9_S_8_/Co_3_S_4_/NF. Except for the ZIF-67/NF (Figure 2e) and S-ZIF-67/NF (Figure 2g) discussed above, MoS_2_/Ni_9_S_8_/NF without ZIF-67 exhibits an open structure composed of nonuniform-sized blocks (Figure 2d). When the Mo source is introduced to the sample, Mo-ZIF-67/NF exhibits a network structure composed of nanosheets (Figure 2f). The above results fully demonstrate that the Mo, S sources, and ZIF-67/NF precursor are essential for generating an amethyst-like rod structure of MoS_2_@Ni_9_S_8_/Co_3_S_4_/NF. The EDS mapping images of MoS_2_@Ni_9_S_8_/Co_3_S_4_/NF indicate a uniform distribution of elemental Mo, Ni, and S (Figure 2h). The structure and composition of MoS_2_@Ni_9_S_8_/Co_3_S_4_/NF were further observed using TEM (Figure 2i). The results reveal that the MoS_2_@Ni_9_S_8_/Co_3_S_4_/NF nanorod comprises a rough surface with a non-hollow structure. An HR-TEM analysis of MoS_2_@Ni_9_S_8_/Co_3_S_4_/NF (Figure 2j) reveals the presence of lattice fringes with 0.42 nm plane spacings belonging to the (201) crystal plane of Ni_9_S_8_ and ones of approximately 0.62 and 0.26 nm corresponding to the (002) and (101) crystal planes of MoS_2_.

**Figure 3:**
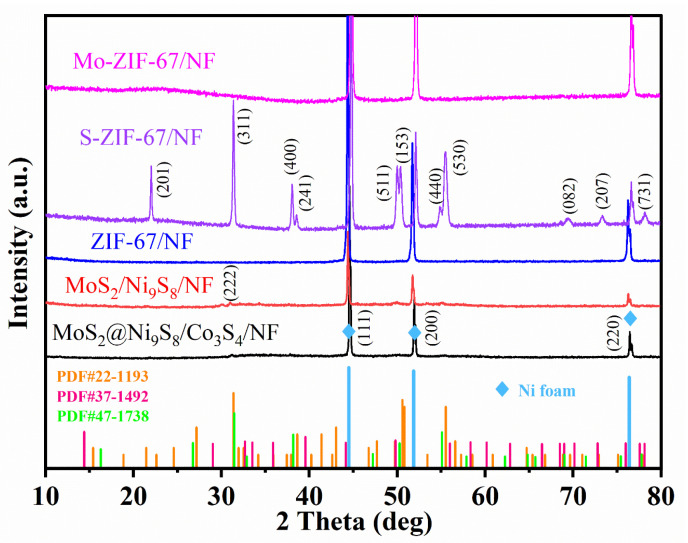
XRD patterns of the as-prepared target and comparison samples.

XRD tests were used to further determine the composition of MoS_2_@Ni_9_S_8_/Co_3_S_4_/NF. As shown in Figure 3, all sample patterns display strong diffraction peaks at 44.5°, 51.8°, and 76.4° corresponding to Ni foam (PDF#87-0712) (111), (200), and (220) crystal planes, respectively, which are derived from the NF substrate. Owing to the strong Ni peak, the diffraction peaks of ZIF-67/NF and Mo-ZIF-67/NF are less obvious. S-ZIF-67/NF exhibits clear diffraction peaks at 31.4°, 38.1°, 50.2°, 55.1°, and 77.8° that correspond to the (311), (400), (511), (440), and (731) crystal planes of Co_3_S_4_ (PDF#47-1738), and the peaks at 21.5°, 38.6°, 50.5°, 55.5°, 69.6°, and 72.9° could be indexed to (201), (241), (153), (530), (082), and (207) crystal planes of Ni_9_S_8_ (PDF#22-1193), respectively. MoS_2_/Ni_9_S_8_/NF and MoS_2_@Ni_9_S_8_/Co_3_S_4_/NF present weak peaks of MoS_2_ (PDF#37-1492) and Ni_9_S_8_ (PDF#22-1193). Thus, to reduce the influence of NF-derived Ni, MoS_2_@Ni_9_S_8_/Co_3_S_4_/NF powder was scraped from the NF for XRD analysis. As shown in Figure S1, the peaks at 14.4°, 32.7°, 35.9°, and 49.8° correspond to the (002), (100), (102), and (105) crystal planes of MoS_2_ (PDF#37-1492) [27], whereas the peaks at 15.4°, 21.5°, 24.6°, 27.2°, 31.3°, 32.5°, 37.9°, 38.6°, 50.8°, 55.5°, and 56.6° belong to the (111), (201), (022), (202), (222), (023), (330), (241), (025), (530), and (531) crystal planes of Ni_9_S_8_ (PDF#22-1193) [50]. The peaks at 16.3°, 31.5°, 38.2°, and 55.1° are indexed to the (111), (311), (400), and (440) planes of Co_3_S_4_ (PDF#47-1738), respectively. The coexistence of the MoS_2_, Ni_9_S_8_, and Co_3_S_4_ crystalline phases confirms that MoS_2_@Ni_9_S_8_/Co_3_S_4_/NF is successfully prepared.

**Figure 4:**
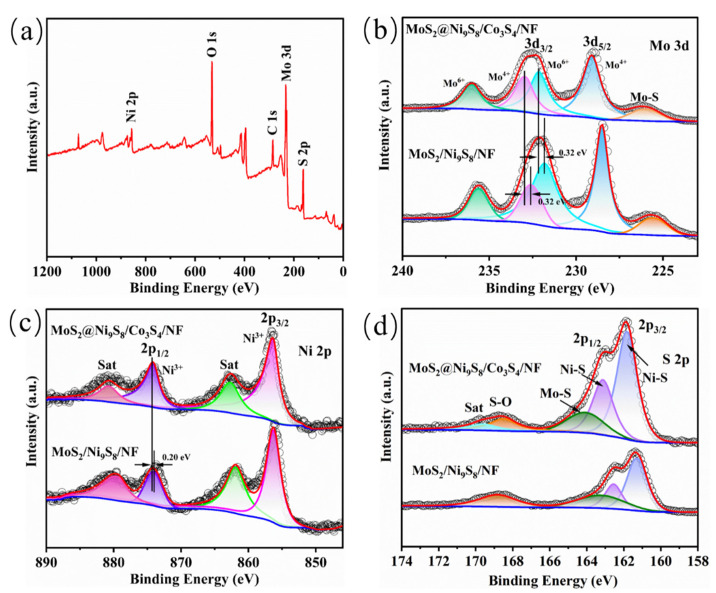
(**a**) XPS full spectrum of the MoS_2_@Ni_9_S_8_/Co_3_S_4_/NF sample and (**b**) Mo 3d, (**c**) Ni 2p, and (**d**) S 2p XPS spectra of MoS_2_@Ni_9_S_8_/Co_3_S_4_/NF and MoS_2_/Ni_9_S_8_/NF.

XPS analysis was performed to characterize the surface element compositions of MoS_2_@Ni_9_S_8_/Co_3_S_4_/NF. The full XPS spectrum of MoS_2_@Ni_9_S_8_/Co_3_S_4_/NF demonstrates the presence of elemental Mo, Ni, and S (Figure 4a). In the Mo 3d spectra of MoS_2_@Ni_9_S_8_/Co_3_S_4_/NF (Figure 4b), the peaks at 229.10 and 232.90 eV correspond to Mo 3d_5/2_ and 3d_3/2_, respectively, which may be assigned to Mo^4+^, whereas those at 232.10 and 235.90 eV can be ascribed to Mo^6+^ [27,51]. The peak at 226.00 eV is assigned to the Mo–S bond [24]. The Ni 2p peaks of MoS_2_@Ni_9_S_8_/Co_3_S_4_/NF at 856.40 and 874.20 eV correspond to Ni 2p_3/2_ and 2p_1/2_, respectively, which are derived from Ni^3+^ [30,52]. Moreover, two satellite peaks (Sat) (Figure 4c) at 862.70 and 880.70 eV are observed. Compared with those observed in the MoS_2_/Ni_9_S_8_/NF without a ZIF-67 spectrum, the Mo 3d and Ni 2p binding energies of MoS_2_@Ni_9_S_8_/Co_3_S_4_/NF exhibit positive shifts of 0.32 and 0.20 eV, respectively, which indicates the process of losing electrons in Mo and Ni elements of MoS_2_@Ni_9_S_8_/Co_3_S_4_/NF [53]. According to previous reports, transition metal sulphides that lose electrons can generate more positive charges, which is conducive to the adsorption of OH^−^ and thus promotes the OER [33,54]. As shown in Figure S2, the peaks at 779.77 and 797.56 eV could correspond to Co 2p_3/2_ and 2p_1/2_, respectively, which may be assigned to Co^3+^ and Co^2+^ [55], and other peaks are ascribed to satellite peaks. The peaks at 161.80 and 163.10 eV are attributed to S 2p_3/2_ and S 2p_1/2_ (Figure 4d), corresponding to the Ni–S bond [54], whereas those at 164.10 and 168.50 eV are ascribed to Mo–S and S–O bonds, respectively [27].

**Figure 5:**
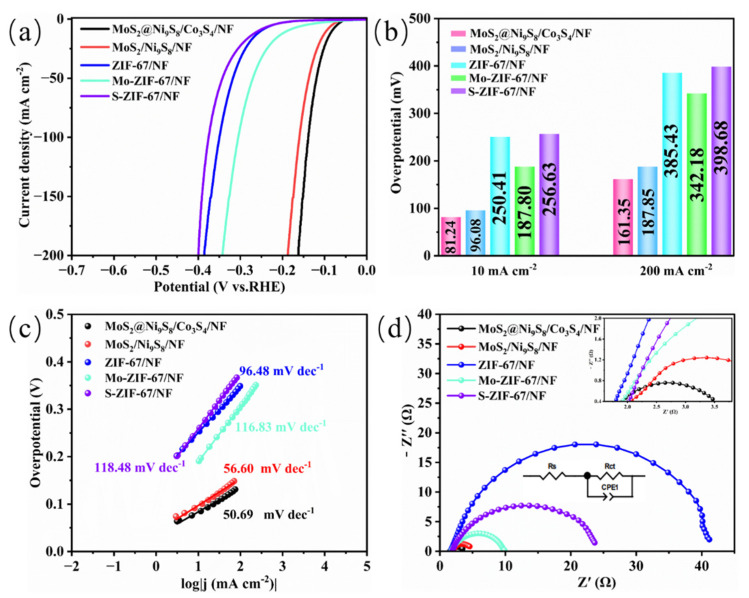
HER performances of the as-prepared samples in 1 M KOH: (**a**) LSV curves, (**b**) overpotential at different current densities, (**c**) Tafel plots, and (**d**) Nyquist plots for the different samples.

The HER performances of the electrocatalysts were tested using a three-electrode system in 1.0 M KOH. As shown in Figure 5a, the linear sweep voltammetry (LSV) curves reveal that MoS_2_@Ni_9_S_8_/Co_3_S_4_/NF has the best HER performance. Figure 5b shows the overpotential values of all the samples at 10 and 200 mA cm^−2^. At a low current density of 10 mA cm^−2^, MoS_2_@Ni_9_S_8_/Co_3_S_4_/NF exhibits the lowest overpotential of 81.24 mV, which is superior to MoS_2_/Ni_9_S_8_/NF (96.08 mV), Mo-ZIF-67/NF (187.80 mV), ZIF-67/NF (250.41 mV), and S-ZIF-67/NF (256.63 mV). The optimal HER performance of MoS_2_@Ni_9_S_8_/Co_3_S_4_/NF originates from the high conductivity and the unique nanorods structure-exposed rich active sites [23,33]. Meanwhile, the improved electronic environment of MoS_2_@Ni_9_S_8_/Co_3_S_4_/NF enhances the adsorption of hydrogen-containing species and accelerates the HER [21,53]. At a high current density of 200 mA cm^−2^, MoS_2_@Ni_9_S_8_/Co_3_S_4_/NF also exhibits superior HER catalytic activity, with an overpotential of 161.35 mV. Comparing the previously reported Mo/Co/Ni-S electrocatalysts for the HER shown in Table S1, the amethyst-like MoS_2_@Ni_9_S_8_/Co_3_S_4_/NF rod electrocatalyst presents the outstanding HER catalytic performance with a lower potential. The Tafel slope was used to study the electrocatalytic kinetics of electrocatalysts. As shown in Figure 5c, The Tafel slopes of MoS_2_@Ni_9_S_8_/Co_3_S_4_/NF, MoS_2_/Ni_9_S_8_/NF, ZIF-67/NF, Mo-ZIF-67/NF, and S-ZIF-67/NF are 50.69, 56.60, 96.48, 116.83, and 118.48 mV dec^−1^, respectively. The lower Tafel slope of MoS_2_@Ni_9_S_8_/Co_3_S_4_/NF indicates more-efficient HER electrocatalytic kinetics [9,23,27]. In addition, the electrocatalytic kinetics were investigated using electrochemical impedance (EIS) analysis. The charge transfer resistance (R_ct_) is related to the electrocatalytic kinetics at the electrolyte–electrode interface. Generally, a smaller R_ct_ represents a higher electron-transfer velocity [30]. Typically, the semicircle diameter in Nyquist plots is positively correlated with the value of R_ct_, and the specific R_ct_ value can be obtained through equivalent circuit fitting [56]. Nyquist plots for all the synthesized samples are shown in Figure 5d, wherein MoS_2_@Ni_9_S_8_/Co_3_S_4_/NF shows the smallest semicircle diameter. The R_ct_ values of MoS_2_@Ni_9_S_8_/Co_3_S_4_/NF, MoS_2_/Ni_9_S_8_/NF, Mo-ZIF-67/NF, and S-ZIF-67/NF are determined as 2.30, 3.27, 8.72, and 23.93 Ω, respectively. ZIF-67/NF presents the highest R_ct_ value (39.49 Ω), which is consistent with the previously reported conclusion that ZIF-67 materials have poor conductivity [8,13,33]. The smallest R_ct_ of MoS_2_@Ni_9_S_8_/Co_3_S_4_/NF reflects the higher electron-transfer velocity and improved conductivity of MoS_2_@Ni_9_S_8_/Co_3_S_4_/NF [21].

**Figure 6:**
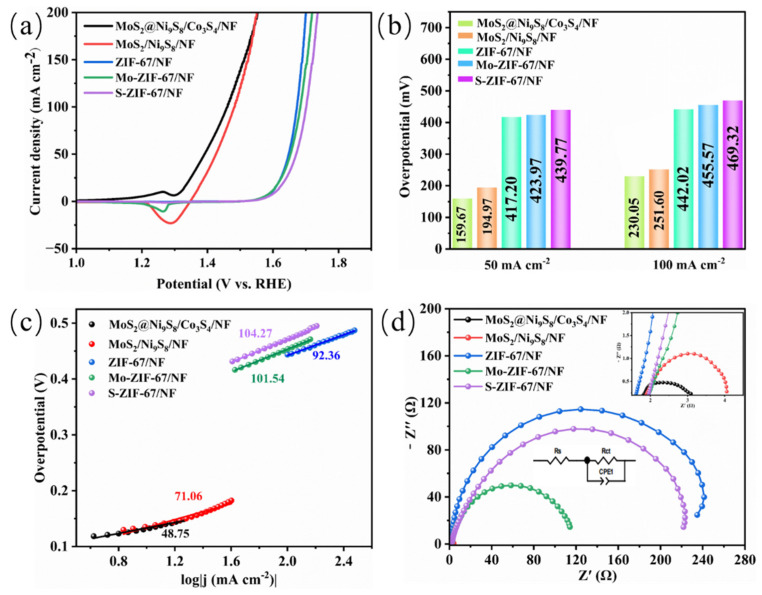
OER performances of the as-synthesized samples in 1 M KOH: (**a**) LSV curves, (**b**) overpotential at different current densities, (**c**) Tafel plots, and (**d**) Nyquist plots for the different samples.

Figure 6a shows the LSV curves of the as-prepared electrocatalysts for the OER. Similar to the HER test results, MoS_2_@Ni_9_S_8_/Co_3_S_4_/NF exhibits superior OER activity. As shown in Figure 6b, the overpotential of MoS_2_@Ni_9_S_8_/Co_3_S_4_/NF is 159.67 mV at 50 mA cm^−2^, which is lower than those of MoS_2_/Ni_9_S_8_/NF (194.97 mV), ZIF-67/NF (417.20 mV), Mo-ZIF-67/NF (423.97 mV), and S-ZIF-67/NF (439.77 mV). Moreover, MoS_2_@Ni_9_S_8_/Co_3_S_4_/NF only requires an overpotential of 230.05 mV to reach a current density of 100 mA cm^−2^. The outstanding OER performance of MoS_2_@Ni_9_S_8_/Co_3_S_4_/NF is related to two factors: on the one hand, the combination of Ni_9_S_8_ and Co_3_S_4_ with OER catalytic activity can produce an effective synergistic effect of components [50,57]. on the other hand, according to the XPS test results, the slight shift of Ni binding energy indicates an improved electronic environment around Ni_9_S_8_, which makes it easier to adsorb OH^−^ and thus promote the OER rate [22,52,53]. Compared with some previously reported electrocatalysts for the OER (Table S2), MoS_2_@Ni_9_S_8_/Co_3_S_4_/NF exhibits competitive OER performance. Figure 6c shows the Tafel plots of the electrocatalysts used for the OER. The Tafel slope of MoS_2_@Ni_9_S_8_/Co_3_S_4_/NF (48.75 mV dec^−1^) is lower than those of MoS_2_/Ni_9_S_8_/NF (71.06 mV dec^−1^), ZIF-67/NF (92.36 mV dec^−1^), Mo-ZIF-67/NF (101.54 mV dec^−1^), and S-ZIF-67/NF (104.27 mV dec^−1^), thus reflecting the faster OER catalytic kinetics of MoS_2_@Ni_9_S_8_/Co_3_S_4_/NF. EIS analysis is performed to further study the electrocatalytic kinetics of the OER (Figure 6d). It can be seen that MoS_2_@Ni_9_S_8_/Co_3_S_4_/NF shows the smallest semicircle diameter. The R_ct_ values of MoS_2_@Ni_9_S_8_/Co_3_S_4_/NF, MoS_2_/Ni_9_S_8_/NF, ZIF-67, Mo-ZIF-67/NF, and S-ZIF-67/NF are determined to be 2.33, 2.80, 248.50, 123.30, and 241.90 Ω, respectively. Notably, the R_ct_ value of MoS_2_@Ni_9_S_8_/Co_3_S_4_/NF is significantly lower than those of the other samples, indicating that MoS_2_@Ni_9_S_8_/Co_3_S_4_/NF possesses the best electron-transfer ability in the OER process [53].

**Figure 7:**
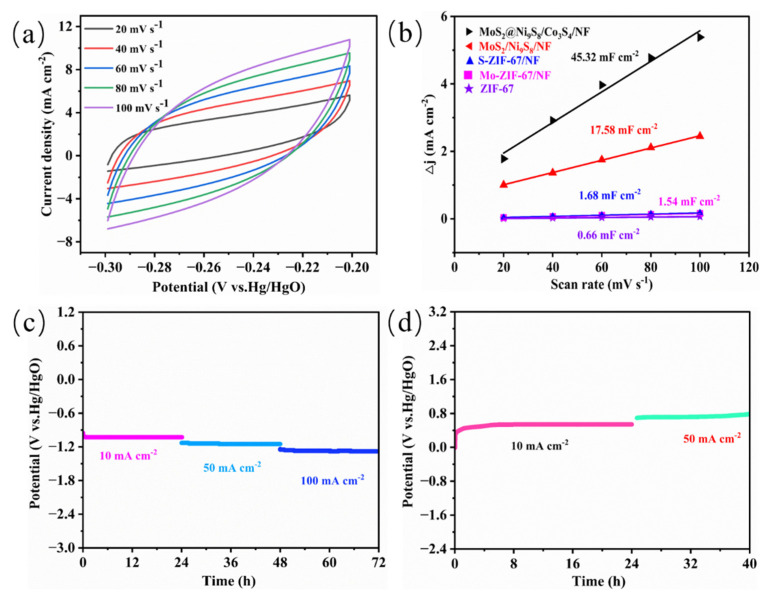
(**a**) CV curves of MoS_2_@Ni_9_S_8_/Co_3_S_4_/NF at different scan rates and (**b**) electrochemical double layer capacitance of the different as-synthesized samples. Performance of MoS_2_@Ni_9_S_8_/Co_3_S_4_/NF during the stability test: (**c**) HER-CP test at 10, 50, and 100 mA cm^–2^; (**d**) OER-CP test at 10 and 50 mA cm^–2^.

Electrochemical active surface area (ECSA) is a vital parameter for evaluating the performance of electrocatalysts [58]. Typically, the ECSA can be determined using the formula:$\;ECSA=C_{dl}/C_{S}$, where ($C_{dl}$) is the double-layer capacitance and $C_{s}$ is the specific capacitance and is generally calculated by using 40.0 μF cm^−2^ [59]. The cyclic voltammetry (CV) curves of the as-prepared samples were measured across the potential range from −0.30 to −0.20 V (vs. Hg/HgO) at scanning speeds of 20–100 mV s^−1^. The CV curves of MoS_2_@Ni_9_S_8_/Co_3_S_4_/NF are shown in Figure 7a, whereas those of MoS_2_/Ni_9_S_8_/NF, ZIF-67/NF, Mo-ZIF-67/NF, and S-ZIF-67/NF are shown in Figure S3a–d, respectively. The $C_{dl}$ can be obtained by fitting the relationship between half the current density at −0.25 V and the scanning speeds. The $C_{dl}$ values of MoS_2_@Ni_9_S_8_/Co_3_S_4_/NF, MoS_2_/Ni_9_S_8_/NF, ZIF-67/NF, Mo-ZIF-67/NF, and S-ZIF-67/NF are determined to be 45.32, 17.85, 0.66, 1.54, and 1.68 mF cm^−2^ (Figure 7b), and the corresponding ECSAs are 1133.0, 446.3, 16.4, 38.5, and 42.0 cm^2^, respectively. Notably, the ECSA of MoS_2_@Ni_9_S_8_/Co_3_S_4_/NF is 2.5 and 69 times larger than those of MoS_2_/Ni_9_S_8_/NF and ZIF-67/NF, respectively. These results demonstrate that the MoS_2_@Ni_9_S_8_/Co_3_S_4_/NF comprising an amethyst-like rod structure can provide a large ECSA and expose abundant active sites. 

Typically, ideal electrocatalysts must not only exhibit high HER and OER activities but also outstanding electrochemical stability. As shown in Figure 7c, during the chronopotentiometry (CP) test for the HER, the potentials of MoS_2_@Ni_9_S_8_/Co_3_S_4_/NF maintain a linear shape after 10, 50, and 100 mA cm^−2^ for 24 h, displaying excellent HER stability. Similarly, Figure 7d reveals the positive OER durability of MoS_2_@Ni_9_S_8_/Co_3_S_4_/NF. The 1000 CV cycles test further evaluated the stability of MoS_2_@Ni_9_S_8_/Co_3_S_4_/NF. As shown in Figure S4a, the LSV curves completely coincide before and after 1000 CV cycles for the HER, with almost no change in the overpotential. However, the overpotential of MoS_2_@Ni_9_S_8_/Co_3_S_4_/NF increases at the same current density for the OER after 1000 CV cycles (Figure S4b). Compared to that of the HER (Figure S5a), the SEM images reveal a more-pronounced degradation of MoS_2_@Ni_9_S_8_/Co_3_S_4_/NF morphology after 1000 CV cycles for the OER (Figure S5b), but the catalyst still attaches to the NF. The relatively weakened OER durability may be derived from the apparent degradation of MoS_2_@Ni_9_S_8_/Co_3_S_4_/NF morphology after 1000 CV cycles, which is due to the violent generation of bubbles during the OER process. Moreover, the samples after 1000 CV cycles were used for the XRD test. It can be seen that no other new diffraction peaks of samples appear after 1000 CV cycles (Figure S6). In the sample after 1000 CV cycles for the HER, the peaks at 31.3°, 37.9°, 40.2°, and 55.5° correspond to the (222), (330), (114), and (530) crystal planes of Ni_9_S_8_ (PDF#22-1193), respectively. The peaks at 33.5°, 35.8°, and 60.1° correspond to the (101), (102), and (008) crystal planes of MoS_2_ (PDF#37-1492), respectively. And in the sample after 1000 cycles for the OER, the peaks at 31.3°, 37.9°, and 55.5° correspond to the (222), (330), and (530) crystal planes of Ni_9_S_8_ (PDF#22-1193), respectively. The peaks at 39.5°, 60.1°, and 85.1° correspond to the (103), (008), and (206) crystal planes of MoS_2_ (PDF#37-1492), respectively. Collectively, these results show that the MoS_2_@Ni_9_S_8_/Co_3_S_4_/NF electrocatalyst possesses outstanding electrochemical stability.

**Figure 8:**
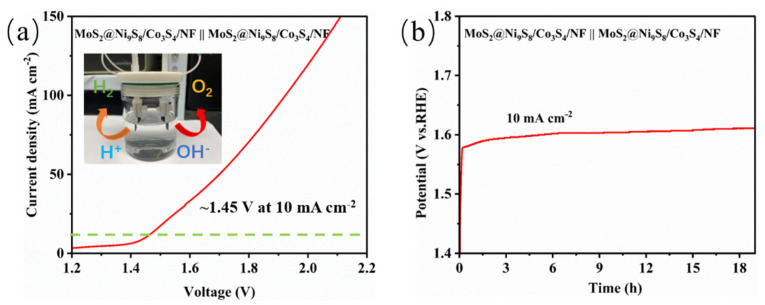
(**a**) LSV curve of MoS_2_@Ni_9_S_8_/Co_3_S_4_/NF in a two-electrode system and (**b**) CP test of MoS_2_@Ni_9_S_8_/Co_3_S_4_/NF at 10 mA cm^–2^.

Owing to the superior electrochemical activities of MoS_2_@Ni_9_S_8_/Co_3_S_4_/NF for the HER and OER, MoS_2_@Ni_9_S_8_/Co_3_S_4_/NF was applied as a bifunctional catalyst for an overall water-splitting test in a two-electrode system. The LSV curve (Figure 8a) shows that the MoS_2_@Ni_9_S_8_/Co_3_S_4_/NF has a low full-cell voltage of 1.45 V at 10 mA cm^−2^, and the cell voltage increases by only 0.03 V after holding at 10 mA cm^−2^ for 19 h (Figure 8b). These results demonstrate that MoS_2_@Ni_9_S_8_/Co_3_S_4_/NF has excellent bifunctional electrocatalytic activity and stability for overall water splitting.

## 4. Conclusions

In summary, we successfully constructed an amethyst-like MoS_2_@Ni_9_S_8_/Co_3_S_4_ rod electrocatalyst using a ZIF-67/NF precursor and a one-step hydrothermal method. By adopting a simple synthesis strategy, MoS_2_@Ni_9_S_8_/Co_3_S_4_/NF possesses high conductivity and numerous active edge sites. Meanwhile, the synergistic effect produced by the composite of MoS_2_, Ni_9_S_8,_ and Co_3_S_4_ further improves the catalytic activity of the electrocatalyst. Therefore, MoS_2_@Ni_9_S_8_/Co_3_S_4_/NF exhibits lower overpotentials and outstanding electrochemical stability in a 1.0 M KOH solution. These results confirm that the as-prepared amethyst-like MoS_2_@Ni_9_S_8_/Co_3_S_4_ rod electrocatalyst possesses excellent bifunctional activity for overall water splitting.

## Data Availability

The data presented in this study are available in this article.

## Supplementary Materials

The following supporting information can be downloaded at: https://www.mdpi.com/article/10.3390/nano13162302/s1, Figure S1: XRD patterns of MoS_2_@Ni_9_S_8_/Co_3_S_4_/NF; Figure S2. Co 2p XPS spectra of MoS_2_@Ni_9_S_8_/Co_3_S_4_/NF; Table S1. Comparison of the HER overpotentials of different electrocatalysts; Table S2. Comparison of the OER overpotentials of different electrocatalysts; Figure S3. CV curves of the samples at different scan rates: (a) MoS_2_/Ni_9_S_8_/NF, (b) ZIF-67/NF, (c) Mo-ZIF-67/NF, and (d) S-ZIF-67/NF; Figure S4. Performance of MoS_2_@Ni_9_S_8_/Co_3_S_4_/NF during the stability test: (a) LSV curves for the HER after 1000 CV cycles, (b) LSV curves for the OER after 1000 CV cycles; Figure S5. SEM images of MoS_2_@Ni_9_S_8_/Co_3_S_4_/NF: (a) after 1000 CV cycles for the HER, (b) after 1000 CV cycles for the OER; Figure S6. XRD patterns of samples after 1000 CV cycles. References [18,20,23,27,30,33,60,61,62] are cited in the supplementary materials.
