# Supplementary material for: Construction of an Amethyst-like MoS2@Ni9S8/Co3S4 Rod Electrocatalyst for Overall Water Splitting
Source: Nanomaterials (Basel). 2023 Aug 10;13(16):2302. doi: 10.3390/nano13162302 (PMC10459789; doi:10.3390/nano13162302)
Supplement: Supplementary file 1 [file nanomaterials-13-02302-s001.zip › nanomaterials-2512671-supplementary.pdf]

Supporting Information

Construction of an amethyst-like $MoS_2@Ni_9S_8/Co_3S_4$ rod electrocatalyst for overall water splitting

Zhen Pei[1], Tengteng Qin[1], Rui Tian[1], Yangxin Ou[1], Xingzhong Guo[1,2*]

1 State Key Laboratory of Silicon and Advanced Semiconductor Materials, School of Materials Science and Engineering, Zhejiang University, Hangzhou 310058, China. E-mail: msewj01@zju.edu.cn(X.G.); 22126097@zju.edu.cn(Z.P.); 12226085@zju.edu.cn(T.Q.); 22026092@zju.edu.cn(R.T.); 22226070@zju.edu.cn(Y.O.)

2 Hangzhou Global Scientific and Technological Innovation Center, Zhejiang University, Hangzhou 311200, People's Republic of China

* Corresponding author, msewj01@zju.edu.cn

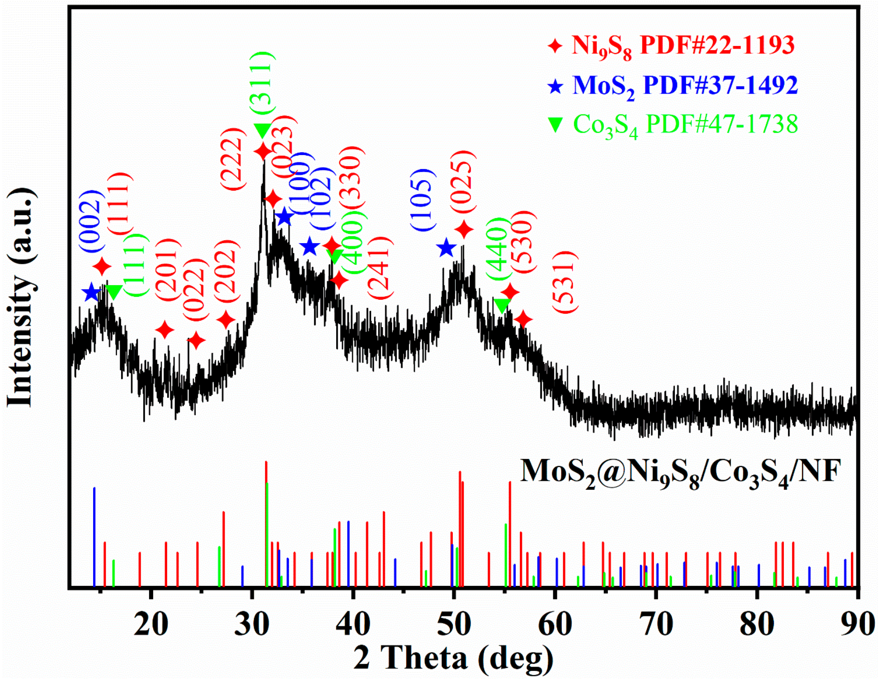

Figure S1. XRD patterns of $MoS_2@Ni_9S_8/Co_3S_4/NF$.

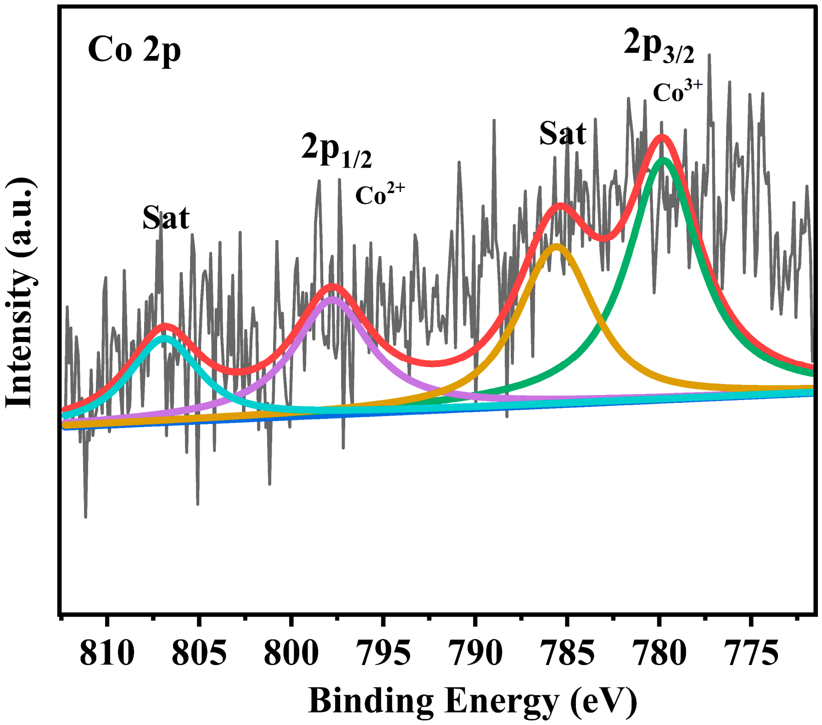

Figure S2. Co 2p XPS spectra of $MoS_2@Ni_9S_8/Co_3S_4/NF$.

Table S1. Comparison of the HER overpotentials of different electrocatalysts.

HER			
Catalyst	η at 10 mA cm^{-2} (mV)	Tafel ($mV\ dec^{-1}$)	Ref
$Co_3O_4@Mo\text{-}Co_3S_4\text{-}Ni_3S_2/NF$	116.0	97.0	[33]
$MoS_2/Co_9S_8/Ni_3S_2/Ni$	113.0	85.0	[30]
Fe-doped Co-Mo-S	105.0	50.3	[23]
Mo,Co-NiS/NF-400	92.0	90.3	[60]
MoS_2/Ni_3S_2	190.0	65.6	[61]
$NiCo_2O_4@NiMo_2S_4$	159.0	53.1	[20]
$Ni_9S_8/CuS/Cu_2O/NF$	146.0	163.0	[18]
**$MoS_2@Ni_9S_8/Co_3S_4/NF$**	**81.2**	**50.7**	**This Work**

Table S2. Comparison of the OER overpotentials of different electrocatalysts.

OER			
Catalysts	η at different current densities (mV)	Tafel ($mV\ dec^{-1}$)	Ref
$Co_3O_4@Mo\text{-}Co_3S_4\text{-}Ni_3S_2/NF$	295.0 (50 mA cm^{-2})	98.0	[33]

CoMoNiS-NF-31	166.0 (10 mA cm⁻²)	58.0	[30]
MoS_2/NiS NCs	271.0 (15 mA cm⁻²)	53.0	[62]
Ni_9S_8/MoS_2@$NiMoO_4$	360.0 (10 mA cm⁻²)	49.8	[27]
Mo,Co-NiS/NF-400	117.0 (10 mA cm⁻²)	68.9	[60]
$NiCo_2O_4$@$NiMo_2S_4$	310.0 (20 mA cm⁻²)	94.5	[20]
**MoS_2@Ni_9S_8/Co_3S_4/NF**	**159.7 (50 mA cm⁻²)**	**48.8**	**This Work**

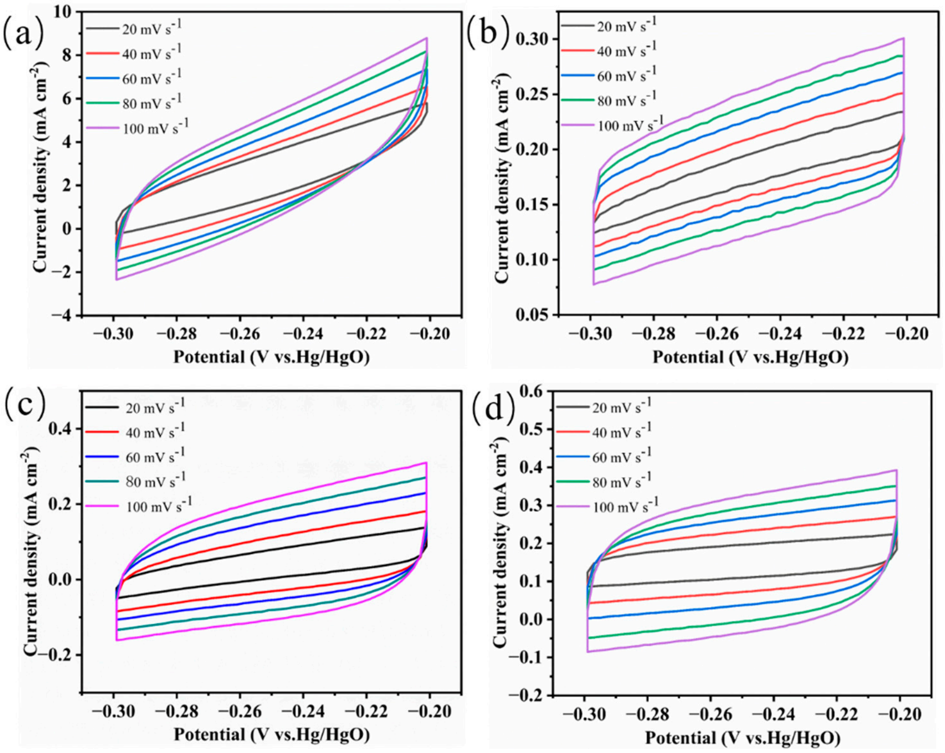

Figure S3. CV curves of the samples at different scan rates: (**a**) MoS_2/Ni_9S_8/NF, (**b**) ZIF-67/NF, (**c**) Mo-ZIF-67/NF, and (**d**) S-ZIF-67/NF.

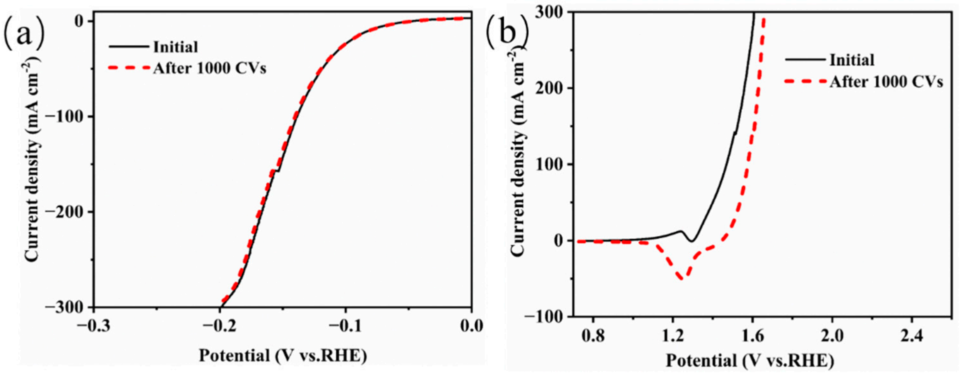

Figure S4. Performance of MoS_2@Ni_9S_8/Co_3S_4/NF during the stability test: (**a**) LSV curves for HER after 1000 CV cycles, (**b**) LSV curves for OER after 1000 CV cycles.

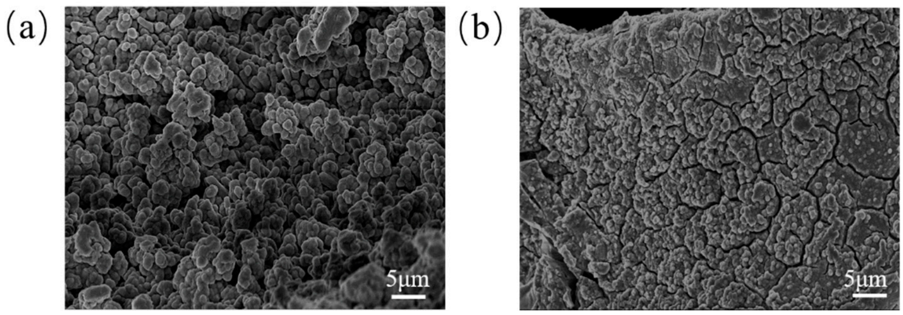

Figure S5. SEM images of MoS_2@Ni_9S_8/Co_3S_4/NF: (**a**) after 1000 CV cycles for HER, (**b**) after 1000 CV cycles for OER.

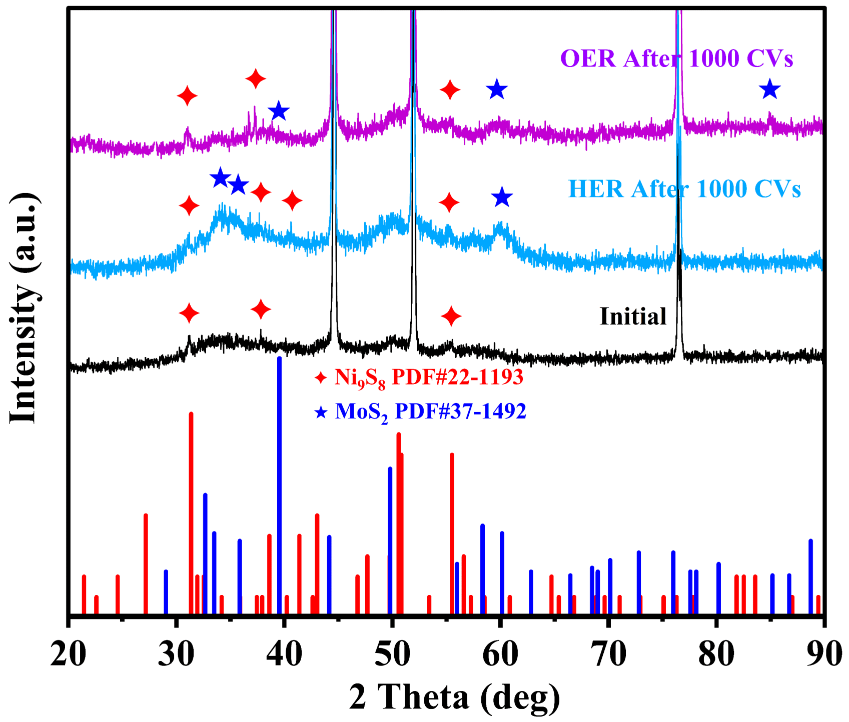

Figure S6. XRD patterns of samples after 1000 CV cycles.

References

33. Wu, Q.; Dong, A.; Yang, C.; Ye, L.; Zhao, L.; Jiang, Q. Metal-organic framework derived Co_3O_4@Mo-Co_3S_4-Ni_3S_2 heterostructure supported on Ni foam for overall water splitting. Chemical Engineering Journal 2021, 413, doi:10.1016/j.cej.2020.127482.
30. Yang, Y.; Yao, H.; Yu, Z.; Islam, S.M.; He, H.; Yuan, M.; Yue, Y.; Xu, K.; Hao, W.; Sun, G., et al. Hierarchical Nanoassembly of MoS_2/Co_9S_8/Ni_3S_2/Ni as a Highly Efficient Electrocatalyst for Overall Water Splitting in a Wide pH Range. Journal of the American Chemical Society 2019, 141, 10417-10430, doi:10.1021/jacs.9b04492.
23. Su, H.; Du, X.; Zhang, X. NiCoP coated on $NiCo_2S_4$ nanoarrays as electrode materials for hydrogen evolution reaction. International Journal of Hydrogen Energy 2019, 44, 30910-30916, doi:10.1016/j.ijhydene.2019.10.046.
60. Wu, C.; Du, Y.; Fu, Y.; Feng, D.; Li, H.; Xiao, Z.; Liu, Y.; Yang, Y.; Wang, L. Mo, Co co-doped NiS bulks supported on Ni foam as an efficient electrocatalyst for overall water splitting

in alkaline media. Sustainable Energy & Fuels 2020, 4, 1654-1664, doi:10.1039/c9se00822e.
61. Narasimman, R.; Waldiya, M.; K, J.; Vemuri, S.K.; Mukhopadhyay, I.; Ray, A. Self-standing, hybrid three-dimensional-porous MoS_2/Ni_3S_2 foam electrocatalyst for hydrogen evolution reaction in alkaline medium. International Journal of Hydrogen Energy 2021, 46, 7759-7771, doi:10.1016/j.ijhydene.2020.12.014.
20. Zhao, D.; Dai, M.; Liu, H.; Chen, K.; Zhu, X.; Xue, D.; Wu, X.; Liu, J. Sulfur-Induced Interface Engineering of Hybrid $NiCo_2O_4$@$NiMo_2S_4$ Structure for Overall Water Splitting and Flexible Hybrid Energy Storage. Advanced Materials Interfaces 2019, 6, 1901308, doi:10.1002/admi.201901308.
18. Wei, D.; Tang, W.; Wang, Y. Hairy sphere-like $Ni_9S_8/CuS/Cu_2O$ composites grown on nickel foam as bifunctional electrocatalysts for hydrogen evolution and urea electrooxidation. International Journal of Hydrogen Energy 2021, 46, 20950-20960, doi:10.1016/j.ijhydene.2021.03.206.
62. Zhai, Z.; Li, C.; Zhang, L.; Wu, H.-C.; Zhang, L.; Tang, N.; Wang, W.; Gong, J. Dimensional construction and morphological tuning of heterogeneous MoS_2/NiS electrocatalysts for efficient overall water splitting. Journal of Materials Chemistry A 2018, 6, 9833-9838, doi:10.1039/c8ta03304h.
27. Chen, L.; Deng, Z.; Chen, Z.; Wang, X. Building Ni_9S_8/MoS_2 Nanosheets Decorated NiMoO4 Nanorods Heterostructure for Enhanced Water Splitting. Advanced Materials Interfaces 2021, 8, 2101483, doi:10.1002/admi.202101483.
